# Caffeine content in newborn hair correlates with maternal dietary intake

**DOI:** 10.1007/s00394-020-02231-2

**Published:** 2020-04-03

**Authors:** Anni Lehtonen, Lauri Uusitalo, Seppo Auriola, Katri Backman, Seppo Heinonen, Leea Keski-Nisula, Markku Pasanen, Juha Pekkanen, Tomi-Pekka Tuomainen, Raimo Voutilainen, Sari Hantunen, Marko Lehtonen

**Affiliations:** 1 Institute of Public Health and Clinical Nutrition, University of Eastern Finland, Kuopio, Finland; 2 Institute of Clinical Medicine, School of Medicine, University of Eastern Finland, Kuopio, Finland; 3 Department of Obstetrics and Gynecology, Kuopio University Hospital, Kuopio, Finland; 4 LC–MS Metabolomics Center, Biocenter, Kuopio, Finland; 5 Department of Pediatrics, Kuopio University Hospital, Kuopio, Finland; 6 Department of Obstetrics and Gynecology, Helsinki University Hospital and University of Helsinki, Helsinki, Finland; 7 School of Pharmacy, University of Eastern Finland, Kuopio, Finland; 8 Department of Public Health, University of Helsinki, Helsinki, Finland; 9 Environmental Health Unit, National Institute for Health and Welfare, Helsinki, Finland

**Keywords:** Caffeine, Diet, Newborn, Pregnancy, Hair, Mass spectrometry, Food safety

## Abstract

**Purpose:**

High-maternal caffeine intake during pregnancy may be harmful for perinatal outcomes and future child health, but the level of fetal cumulative exposure has been difficult to measure thus far. Here, we present maternal dietary caffeine intake during the last trimester and its correlation to caffeine content in newborn hair after birth.

**Methods:**

Maternal third trimester diets and dietary caffeine intake were prospectively collected in Kuopio Birth Cohort (KuBiCo) using a 160-item food frequency questionnaire (*n* = 2840). Newborn hair was collected within 48 h after birth and analyzed by high-resolution mass spectrometry (HRMS) for caffeine (*n* = 316). Correlation between dietary caffeine intake and neonatal hair caffeine content was evaluated from 203 mother–child pairs.

**Results:**

Mean dietary caffeine intake was 167 mg/days (95% CI 162–172  mg/days), of which coffee comprised 81%. Caffeine in the maternal diet and caffeine content in newborn hair correlated significantly (*r* = 0.50; *p* < 0.001). Older, multiparous, overweight women, and smokers had the highest caffeine levels in the maternal diet, as well as in their newborn babies’ hair.

**Conclusion:**

Caffeine exposure, estimated from newborn hair samples, reflects maternal third trimester dietary caffeine intake and introduces a new method to assess fetal cumulative caffeine exposure. Further studies to evaluate the effects of caffeine exposure on both perinatal and postnatal outcomes are warranted, since over 40% of pregnant women consume caffeine more than the current suggested recommendations (European Food Safety Association, EFSA recommendations).

## Introduction

Caffeine, the world’s most widely used central nervous system stimulant, is present in many drinks and foods consumed during pregnancy. In Europe, the mean daily caffeine intake is usually 100─300 mg during pregnancy, while many women consume a greater amount of caffeine [1]. The dietary caffeine intake is generally high in Finland, being the highest in the world (9.9 kg coffee/person/year 2018) and twice the European Union countries average [2]. According to the European Food Safety Authority (EFSA, 2015), pregnant women should limit daily caffeine intake to 200 mg per day. The recommendation is based on the prospective cohort studies, which have shown a dose–dependent association between caffeine intake during pregnancy and the risk of weight-related outcomes, such as fetal growth restriction and the birth of a small for gestational age (SGA) child [3, 4]. A high intake was also associated with other adverse obstetric outcomes, such as pregnancy loss and stillbirth [5–8]. However, studies on birth outcome show conflicting findings: the long-term effects of caffeine on the developing fetus are still unknown [9], including that the level of fetal exposure is difficult to measure.

Newborn hair samples collected shortly after birth provide new insight for studying long-term cumulative fetal exposures. Fetal scalp hair begins to grow after the 18th to 20th gestational week, and grows approximately one cm per month [10]. Most exposure agents, including caffeine and paraxanthine, pass through the placenta, affect fetal circulation, and stably incorporate into the growing fetal hair. Thus, newborn hair represents cumulative fetal exposure during the third trimester of pregnancy, similarly to how adult hair samples represent cumulative exposure during the preceding months in adults [11, 12]. Caffeine is mainly metabolized to paraxanthine and other isomers as theobromine and theophylline in the liver by the CYP1A2 enzyme. Paraxanthine has similar biological activities as caffeine and it is not found in the plants naturally and is thus always a metabolite of caffeine [13]. Caffeine and paraxanthine content in hair can be measured by high-resolution mass spectrometry (HRMS), which provides superior sensitivity and specificity, as compared to other methods such as immunoassays [14].

In this study, we prospectively evaluated maternal dietary caffeine, with a 160-item food frequency questionnaire (FFQ) for the last trimester of pregnancy and measured newborn hair caffeine content: this represented cumulative fetal caffeine exposure for the last trimester of pregnancy. The aims of this study were to assess maternal dietary caffeine in the third trimester and evaluate whether dietary caffeine levels correlate with newborn hair caffeine and paraxanthine content. In addition, we evaluated whether maternal or pregnancy characteristics played a role in maternal dietary caffeine intake and measured newborn’s hair caffeine levels.

## Subjects and methods

This study used data from the Kuopio Birth Cohort, KuBiCo [15]. Briefly, participants in KuBiCo were recruited at the prenatal clinics by primary personnel at outpatient health care centers (majority > 90% during the first trimester) from the pregnant women of Northern Savo, Finland. The only inclusion criterion in the study is that the participant is expected to give birth after the 22nd gestational week in Kuopio University Hospital, although there are no specific exclusion criteria. Participation is voluntary, as women give informed consent during recruitment. We excluded those with multiple gestations and stillbirths. The Research Ethics Committee of the Hospital District of Central Finland in Jyväskylä reviewed and approved the KuBiCo plan in 2011.

### Maternal dietary caffeine assessment

Maternal diet and caffeine intake were estimated from a total of 2840 pregnancies in KuBiCo between June 2012 and July 2018, using FFQ during the last trimester of gestation (weeks 28–40). The FFQ used was a slightly modified version from the Kuopio Breast Cancer Study, where its validity and reliability were described [16]. The FFQ was designed to cover the whole diet during the preceding three months. To reflect the increased selection of food items available, the number was increased to 160: for instance, the use of energy drinks and bars.

Food items were presented as 13 subgroups, including dairy and grain products, vegetables, fruits, and berries. The portion sizes were fixed and specified with natural units (e.g., slice and glass) or grams. The FFQ comprises a food list of 160 food items with 9 frequency response options, ranging from 'never' to '6 or more times per day.' The food consumption and nutrient intakes were calculated by multiplying the frequency of food consumption by portion size (g) to obtain consumption of each listed food item as representing the average daily consumption. The output provided more than 60 nutrients and 100 food groups.

The participants completed the web-based FFQ and only fully completed FFQ was returned to the study database. Average daily food, nutrients, and energy intake, were calculated in 2018, using the Finnish national food composition (Fineli) database. In the KuBiCo study, FFQ also produced automatized personal feedback to all study subjects within 24 h after they returned the FFQ via the Internet.

The caffeine intake was estimated by summarizing the daily consumption of coffee (caffeine 70 mg/100 mL), cola drinks (20 mg/100 mL), tea (15 mg/100 mL), energy drinks (32 mg/100 mL), cocoa (3 mg/100 mL), and chocolate (30 mg/100 g).

Among 2840 pregnant women, the following descriptive characteristics were collected: maternal age, BMI (kg/m^2^) in the first trimester, weight gain during pregnancy, smoking during and before pregnancy, parity, duration of gestation at birth, and fetal sex. These parameters were used as comparative covariates (characteristics) among distributions of maternal dietary caffeine.

### Newborn hair caffeine content

We collected 316 hair samples after birth (< 48 h) from 316 singleton newborns at Kuopio University Hospital (between May 2017 and March 2018). Hair samples were cut with fine scissors as close to the scalp as possible, from the occipital region, and stored in aluminum foil at room temperature (21 ± 2 °C) [17] for 1 to 11 months prior to mass spectrometric analysis.

Prior to the analysis, the hair sample (approximately 5 mg) was washed with 1.0 ml of isopropanol (30 s) to remove all external contaminants. Isopropanol was removed with a Pasteur pipette from the sample, which evaporated to the dryness level in the context of nitrogen (N-EVAP 112, Organomation Associates, Inc., Berlin, MA, USA). The residue was extracted with a normalized volume of methanol (1 mg equals 250 µl of methanol) in a shaker (Heidolph Multi Reax, Heidolph Instruments GmbH & Co, Schwabach, Germany) for 20 h at room temperature (+ 21 ± 2 °C). After incubation, the methanol extract was centrifuged (8000 rpm, 10 min, + 21 °C) and transferred to the 96-well plate for mass spectrometric analysis. Sample order (for analysis of the samples) was randomized. Aliquots of 2 μL were taken from 231 hair samples, pooled, and used as the quality control (QC) sample. During the sample sequence, QC samples were injected in the beginning of the sequence and after every 12 samples.

Targeted metabolite profiling analysis was carried out at the LC–MS Metabolomics Center (Biocenter Kuopio, University of Eastern Finland). The analysis was conducted with an ultra-high-performance liquid chromatograph (Vanquich Flex UHPLC System, Thermo Scientific, Bremen, Germany), coupled online to an HRMS (Q Exactive Focus, Thermo Scientific, Bremen, Germany). The samples were analyzed using a reversed-phase (RP) chromatographic technique. The sample solution (2 µl) was injected onto a column (Zorbax Eclipse XDBC18, 2.1 × 100 mm, 1.8 μm, Agilent Technologies, Palo Alto, CA, USA), which was kept at 40 °C. Mobile phases, delivered at 400 μL/min, consisted of water (eluent A) and methanol (eluent B), each containing 0.1% (v/v) of formic acid. The following gradient profile was used: 0–10 min: 2–100% B, 10–14.50 min: 100% B, 14.50–14.51 min: 100–2% B, and 14.51–20 min: 2% B. The sample tray was 10 °C during these analyses.

Mass spectrometry was equipped with a heated electrospray ionization (ESI), while the positive ionization mode was used to acquire the data. The following ESI source settings were utilized: spray voltage (3.5 kV), sheath gas (40), auxiliary gas (10), and sweep gas (2) (flow rates as arbitrary units for ion source). The capillary temperature and the probe heater temperature were set at 300 °C. The S-lens RF level was set to 50 V. A full scan range from 120 to 1100 (*m*/*z*) was used with the resolution of 70, 000 (m/Δm, full width at half maximum of 200 μ). Automatic injection time was used, while automated gain control (AGC) was targeted at 1,000,000 ions. The detector was calibrated before the sample sequence and operated at high mass accuracy (< 2 ppm).

For MS/MS experiments, the Q-Exactive spectrometer was used with the same source parameters and chromatography conditions as above. Two scan events were used: (i) an MS scan with mass resolution power, AGC target and maximum injection time set to 35,000 (m/Δm, full width of half maximum at 200 μ), 1,000,000 ions, and automatic, respectively, and (ii) an MS/MS scan (in HCD mode) at a normalized collision energy, ranging from 20 to 40%, depending on the molecule, with a mass resolution power, AGC target, maximum injection time, isolation window set to 17,500 (m/Δm, full width at half maximum at 200 μ), 50,000, automatic, and 1.5 *m*/*z*, respectively. Loop count was 3, apex trigger 0.2–3 s, and dynamic exclusion 15 s.

TraceFinder 4.1. software (Thermo Scientific, Bremen, Germany) was used for data processing and visualization. The identification of caffeine and its metabolites (i.e., paraxanthine, theobromine, and theophylline) was based on the accurate mass and isotope information, as well as product ion spectra (MS/MS). In addition, caffeine and paraxanthine were identified with authentic standard compounds by comparison of retention times in standards and samples. The QC samples were used to monitor the stability and functionality of the system throughout the sample sequence. The relative standard deviation of the peak areas in QC samples (*n* = 50) for caffeine, paraxanthine, theobromine, and theophylline were 9.3%, 17.8%, 10.6%, and 12.8, respectively. The range of linearity was inspected with pure standard solutions with a concentration range of 2–2000 nM, corresponding to 0.1–97 ng/mg of hair. The coefficient of determination of the calibration was 0.996, which shows good linearity of the method.

### Data analysis

Maternal dietary caffeine intakes were expressed as mean values, with 95% confidence intervals, and the proportions of caffeine sources were expressed as mean values, with the percentage of total caffeine intake. The measured newborn hair caffeine content was reported as nanograms of caffeine per milligrams of a hair sample (ng/mg) and expressed as the median and mean with 95% confidence intervals (95% CI). *p* values were calculated by comparing distribution across groups by independent samples: the Mann–Whitney *U* test (2 groups) or the Kruskal–Wallis test was used (≥ 3 groups). Spearman’s rank-order correlation was used to estimate the strength and direction of the associations. A multiple linear regression model was applied to explore which factors were independently associated with newborn hair caffeine levels. *β* values as standardized coefficients were used to compare the strength of associations between the studied factors. The results with a *p* value < 0.05 were considered statistically significant.

## Results

### Maternal dietary caffeine

The descriptive value of maternal dietary caffeine intake and proportion of caffeine sources are shown in Tables 1 and 2. During the last trimester of pregnancy, the mean daily caffeine intake was 167 mg (95% CI 162–172 mg) among 2840 pregnant women. The overweight, smoking, multiparous, and oldest women had the highest caffeine levels in their diet. Approximately 78–85% of daily caffeine was obtained from coffee, 8–13% from caffeinated cola drinks, 3–8% from tea, 2–3% from cocoa and chocolate, and 0–1% from energy drinks.

**Table 1:** Dietary caffeine intake in third trimester among 2840 pregnant women in relation to maternal and fetal characteristics

Characteristics	Count (%)	Mean caffeine intake (mg/days)	95% CI for mean (mg/days)	*p* value
Maternal age (years)				< 0.001
< 28	835 (30)	151	143–160	
28–32	922 (32)	169	161–177	
> 32	1083 (38)	178	170–186	
Maternal BMI (kg/m^2^)^a^				0.016
< 25	1729 (62)	161	155–167	
25–30	631 (23)	181	170–192	
> 30	404 (15)	171	157–185	
Maternal weight gain (kg)				0.746
< 12	1139 (39)	165	158–172	
12–15	866 (31)	166	157–174	
> 15	835 (30)	172	163–181	
Smoking status^b^				< 0.001
Not smoking	2300 (81)	155	150–160	
Quit smoking	413 (15)	213	200–226	
Smoking	93 (3)	265	231–300	
Not known	34 (1)	188	151–226	
Parity				< 0.001
Nulliparous	1371 (48)	150	144–157	
Primiparous	869 (31)	177	168–185	
Multiparous	600 (21)	192	181–203	
Duration of gestation at birth (days)				0.027
< 259	154 (5)	153	133–173	
259–279	1257 (45)	166	159–173	
> 279	1429 (50)	170	163–177	
Fetal sex				0.925
Male	1461 (51)	167	160–174	
Female	1379 (49)	167	161–74	
Total of population	2840 (100)	167	162—172	

^a^*n* = 2764; ^b^"not smoking” were defined as not smoking or passive smoking, "quit smoking" were defined as quit smoking before pregnancy or at first trimester of pregnancy, "smoking" were defined as smoking during pregnancy or quit at second or third trimester of pregnancy; *BMI* body mass index at first trimester; *p* values were calculated by comparing distributions across groups by independent samples Mann–Whitney *U* test (‘Fetal sex’) or Kruskal–Wallis test (other characteristics).

**Table 2:** Mean dietary caffeine proportions by different caffeine sources in relation to maternal and fetal characteristics in third trimester among 2840 pregnant women

Characteristics	Coffee, mg/days (%)	Cola drinks, mg/days (%)	Tea, mg/days (%)	Energy drinks, mg/days (%)	Cocoa and chocolate mg/days (%)	Total, mg/days (%)
Maternal age (years)						
< 28	118 (78)	18 (12)	10 (6)	1 (1)	4 (3)	151 (100)
28–32	140 (83)	14 (8)	12 (7)	0 (0)	3 (2)	169 (100)
> 32	145 (81)	14 (8)	15 (8)	1 (1)	3 (2)	178 (100)
Maternal BMI (kg/m^2^)^a^						
< 25	132 (82)	12 (8)	13 (8)	0 (0)	4 (2)	161 (100)
25–30	144 (80)	18 (10)	13 (7)	2 (1)	4 (2)	181 (100)
> 30	135 (79)	23 (13)	9 (5)	1 (1)	3 (2)	171 (100)
Maternal weight gain (kg)						
< 12	132 (80)	16 (10)	12 (7)	1 (1)	3 (2)	164 (100)
12–15	136 (82)	13 (7)	13 (8)	1 (1)	3 (2)	166 (100)
> 15	140 (82)	16 (9)	12 (7)	0 (0)	4 (2)	172 (100)
Smoking status^b^						
Not smoking	124 (80)	14 (9)	13 (8)	1 (1)	3 (2)	155 (100)
Quit smoking	179 (84)	20 (9)	9 (4)	1 (1)	4 (2)	213 (100)
Smoking	225 (85)	25 (9)	8 (3)	3 (1)	4 (2)	265 (100)
Not known	154 (82)	18 (10)	12 (6)	0 (0)	4 (2)	188 (100)
Parity						
Nulliparous	119 (79)	15 (10)	12 (8)	1 (1)	3 (2)	150 (100)
Primiparous	144 (81)	15 (9)	13 (7)	2 (1)	3 (2)	177 (100)
Multiparous	161 (84)	16 (8)	12 (6)	0 (0)	3 (2)	192 (100)
Duration of gestation at birth (days)						
< 259	123 (81)	13 (9)	12 (8)	0 (0)	3 (2)	153 (100)
259–279	134 (81)	16 (10)	13 (8)	1 (1)	4 (2)	166 (100)
> 279	139 (82)	14 (8)	12 (7)	1 (1)	3 (2)	170 (100)
Fetal sex						
Male	134 (80)	15 (9)	13 (8)	1 (1)	3 (2)	167 (100)
Female	137 (82)	15 (9)	12 (7)	0 (0)	3 (2)	167 (100)
Total of population (*n* = 2840)	136 (81)	15 (9)	12 (7)	1 (1)	3 (2)	167 (100)

^a^*n* = 2764; ^b^"not smoking” were defined as not smoking or passive smoking, "quit smoking" were defined as quit smoking before pregnancy or at first trimester of pregnancy, "smoking" were defined as smoking during pregnancy or quit at second or third trimester of pregnancy; *BMI* body mass index at first trimester

Table 3 shows four different grades of maternal daily dietary caffeine intake (from low to very high) in the last trimester. Among all women, 23% consumed less than 50  mg/days of caffeine (low level), 36% consumed an average level (50–199  mg/days), 31% consumed a high level (200–299  mg/days), and 10% consumed a very high level (≥ 300  mg/days). In caffeine consumers throughout pregnancy, 41% consumed more than EFSA recommends (≥ 200  mg/days).

**Table 3:** The proportions of dietary caffeine intake in third trimester among 2840 pregnancies by four grades of daily intake

Grade of daily caffeine intake	Count (%)
Low (< 50 mg/days)	655 (23)
Average (50–199 mg/days)	1015 (36)
High (200–299 mg/days)	883 (31)
Very high (≥ 300 mg/days)	287 (10)
Total	2840 (100)

### Newborn hair caffeine and paraxanthine

The descriptive value of caffeine levels in 316 newborn hair samples is shown in Table 4. The mean caffeine content in newborn hair was 28 ng/mg (95% CI 25–30 ng/mg). The newborns for older or multiparous women had significantly higher hair caffeine levels, as compared to those born to younger or nulliparous women, respectively.

**Table 4:** Caffeine levels in neonatal hair among 316 newborns in relation to maternal and fetal characteristics

Characteristics	Count (%)	Median (ng/mg)	Mean (ng/mg)	95% CI for mean (ng/mg)	*p* value
Maternal age (years)					0.015
< 28	103 (33)	18.5	23.0	19.2–26.8	
28–33	110 (35)	23.4	29.5	25.4–33.7	
> 33	103 (33)	25.3	30.2	25.9–34.4	
Maternal BMI (kg/m^2^)^a^					0.456
< 25	188 (61)	21.0	27.7	24.4–30.9	
25–30	70 (23)	24.7	28.8	24.5–33.1	
> 30	51 (17)	23.6	28.0	21.9–34.0	
Maternal weight gain (kg)^b^					0.354
< 12	114 (36)	23.8	27.4	23.6–31.2	
12–15	93 (30)	22.1	30.4	25.6–35.2	
> 15	107 (34)	19.7	25.5	21.6–29.3	
Smoking status^b^					0.610
Not smoking	254 (81)	21.9	27.1	24.5–29.7	
Quit smoking	51 (16)	22.1	30.4	24.0–36.8	
Smoking	9 (3)	23.7	28.6	11.5–45.8	
Parity					< 0.001
Nulliparous	155 (49)	16.7	19.6	17.1–22.1	
Primiparous	88 (28)	29.9	34.1	29.2–39.1	
Multiparous	73 (23)	31.3	36.7	31.3–42.2	
Duration of gestation at birth (days)					0.196
< 259	14 (5)	24.7	26.3	14.8–37.7	
259–279	149 (47)	25.2	29.3	25.9–32.7	
> 279	153 (48)	19.1	26.1	22.6–29.6	
Fetal sex					0.252
Male	165 (52)	22.6	28.5	25.3–31.8	
Female	151 (48)	21.9	26.6	23.1–30.1	
Total of population	316 (100)	22.0	27.6	25.3–30.0	

^a^*n* = 309; ^b^*n* = 314; "not smoking” were defined as not smoking or passive smoking, "quit smoking" were defined as quit smoking before pregnancy or at first trimester of pregnancy, "smoking" were defined as smoking during pregnancy or quit at second or third trimester of pregnancy; *BMI* = body mass index at first trimester; *p* values calculated by comparing distributions across groups by independent samples Mann–Whitney *U* test (‘Fetal sex’) or Kruskal Wallis test (other characteristics)

A multiple linear regression model was created to predict newborn hair caffeine content based on maternal dietary caffeine, age, parity, BMI, weight gain during the pregnancy, smoking status, fetal sex, and gestational age at birth [*F*(8,193) = 9.2, *p* < 0.001, *R*^2^ = 0.28]. Maternal caffeine intake and parity were significant predictors (*β* = 0.37; *p* < 0.001 and *β* = 0.23; *p* < 0.001, respectively), while the other variables in the model were not.

**Fig. 1:**
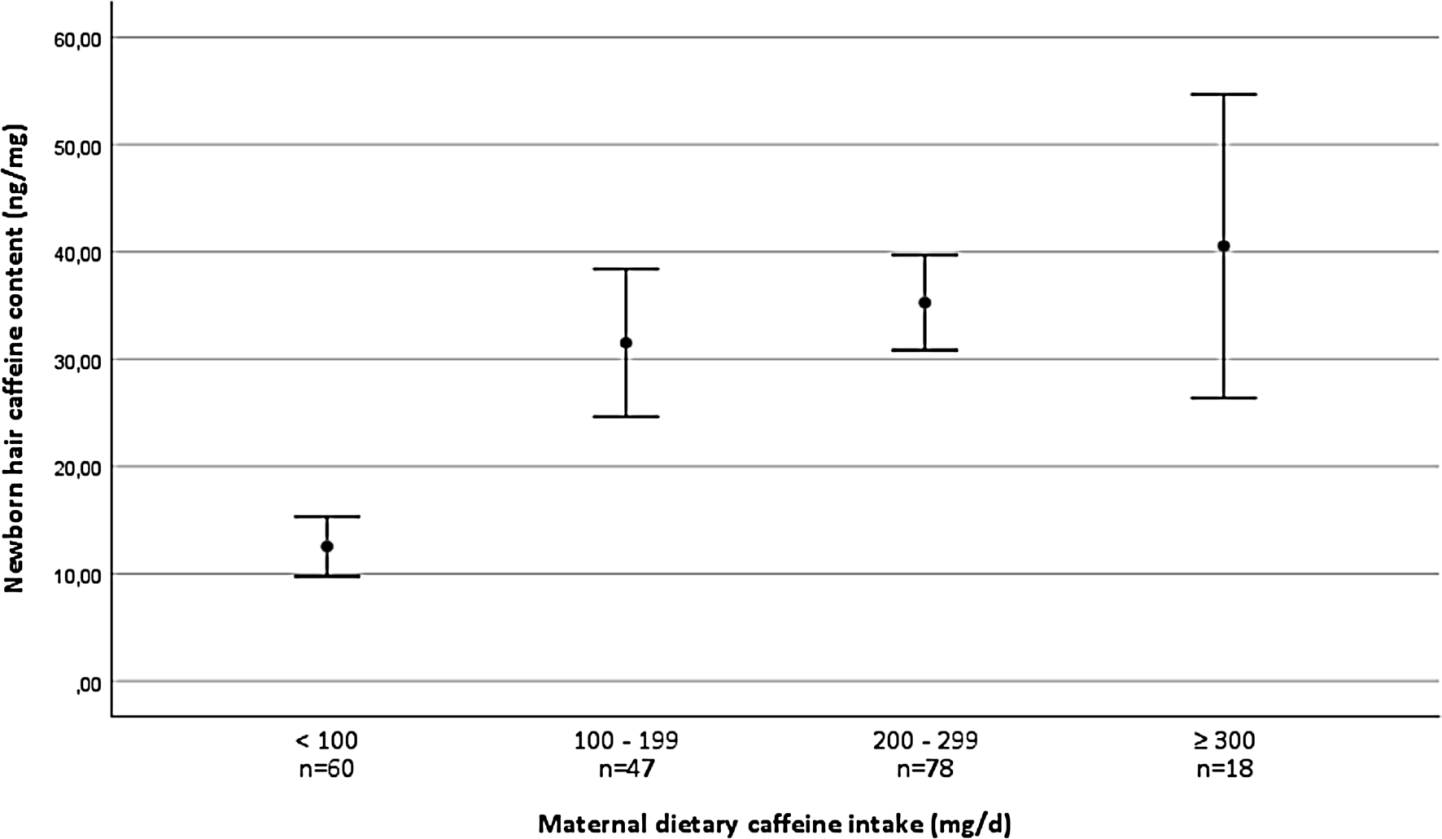
Relationship between maternal dietary caffeine intake and measured newborn hair caffeine content among 203 mother–child pairs. Mothers were grouped in four grades by mean caffeine daily intake (mg/days) and compared to these groups newborns’ hair caffeine content means and 95% CI. Newborn hair caffeine content was expressed as nanograms of caffeine measured per milligram of hair sample (ng/mg)

The relationship between maternal dietary caffeine and newborn hair caffeine levels among 203 mother–child pairs is shown in Fig. 1. Caffeine in the maternal diet correlated positively with caffeine and paraxanthine content in newborn hair separately and combined (caffeine: *r* = 0.49; *p* < 0.001; paraxanthine: *r* = 0.48; *p* < 0.001; combined *r* = 0.50; *p* < 0.001). The correlation between caffeine and paraxanthine contents in the newborn hair samples was strong (*r* = 0.78; *p* < 0.001) and the paraxanthine/caffeine mean ratio was 0.102 (95% CI 0.095–0.110). In total, 62% of the women who reported caffeine use of less than 100  mg/days (FFQ) found their newborn child's hair caffeine level in the lowest quartile (< 25th percentile of all samples). Furthermore, 76% of the women who reported intake equal to or more than 200  mg/days caffeine in their diet, found their newborn child's hair caffeine level to be in the highest quartile (> 75th percentile).

## Discussion

To the best of our knowledge, this study is the first to show that caffeine and its major metabolite paraxanthine are measurable in newborn hair by the HRMS method, and that these levels correlate positively with the approximated maternal caffeine intake during the last trimester of pregnancy. First, this confirms that caffeine diffuses from maternal to fetal circulation through the placenta and is incorporated into the growing fetal hair [8, 12]. This indicates that long-term cumulative fetal exposure can be measured in a newborn’s hair. Second, the strong correlation between caffeine content in the maternal diet and newborn hair indicates that FFQ and approximation of maternal dietary caffeine intake can serve as a valid method to evaluate fetal cumulative caffeine exposure, even when requesting pregnant women to report their diet based on a long-term perspective. Third, we were surprised that more than 40% of pregnant women consumed caffeine more than EFSA recommends. Older and overweight mothers, smokers and multiparas consumed higher amounts of caffeine than the women in comparison groups. The previously reported finding that smokers have increased amounts of caffeine in their diet [3, 18] was observed also in our study. A common dietary change during pregnancy is to decrease caffeine intake for the health status of the fetus, and nulliparas are more likely to decide on healthier choices in their diet than primiparas or multiparas are [19]. We confirm this finding, as we observed that the level of caffeine exposure on the fetus was strongly dependent on maternal parity.

The KuBiCo participants represent the Finnish pregnant women and newborns [15], as the study data were collected year-round with standardized, supervised methods. We used high-accuracy mass spectrometry with superior sensitivity and specificity for hair caffeine measurements. The standards prepared in pure solvents could report hair caffeine values, which were within the linear range of the method.

We show that the most consumed source of caffeine in the maternal diet was coffee (over 80% percent of the reported caffeine intake). In caffeine-containing drinks, the amount of caffeine depends on the brand, the type of beans and leaves, preparation, serving, and cup size. These factors may have caused bias, considering our approximation of maternal dietary caffeine [20]; this can be considered a weakness of our study. Also, given the nature of FFQ, there may be recall bias in the reported consumption of caffeine-containing products by study participants. In hair analyses it is important to notice that the hair sample taken only from one region of the head may contribute to a measurement error and further research should consider whether one hair lock is consistently representative [21].

In conclusion, measured newborn hair caffeine content correlated well with maternal dietary caffeine intake during the last trimester of pregnancy, representing fetal cumulative caffeine exposure. Newborns of older, overweight, multiparous, and smoking women had the highest exposure to caffeine, likely explained by differences in maternal diets. Further studies are warranted to evaluate the effect of the measured caffeine exposure on perinatal and future child health outcomes, since over 40% of pregnant women consume more caffeine than the current suggested recommendations.

## Conclusion

Newborn hair caffeine and paraxanthine content, measured by HRMS, correlated significantly with maternal dietary caffeine intake during the last trimester of pregnancy; thus, newborn hair caffeine and paraxanthine content reflects fetal cumulative caffeine exposure. The newborns of older, overweight, multiparous, and smoking women had the highest exposure to caffeine in utero, which can be explained by differences in maternal diets. Further studies are warranted to evaluate the effect of caffeine exposure on perinatal and future child health outcomes; according to our results, over 40% of pregnant women consume caffeine more than the current suggested recommendations.
